# Mental health literacy moderates the association between anxiety and depressive symptoms among Chinese young adults: a population-based cross-sectional study

**DOI:** 10.3389/fpubh.2026.1872627

**Published:** 2026-10-16

**Authors:** Xuhua Chen, Wenqing Rao, Duanhua Xiong, Jiawu Ji

**Affiliations:** 1 Institute of Information Management, Fuzhou University, Fuzhou, China; 2 Fuzhou Neuropsychiatric Hospital Affiliated to Fujian Medical University, Fuzhou, China

**Keywords:** anxiety, depression, influencing factors, mental health literacy, moderation, young adults

## Abstract

**Background:**

Mental health literacy (MHL) plays an important role in the recognition, prevention, and management of mental disorders, but its current status among Chinese young adults and its potential moderating role in the association between anxiety and depression remain underexplored. This study examined MHL among young adults in China, identified its influencing factors, and tested whether MHL moderates the relationship between anxiety and depressive symptoms.

**Methods:**

A cross-sectional survey was conducted in Fujian Province, China, using multistage stratified cluster random sampling. A total of 2,164 young adults aged 18–35 years were included in the analysis. Measurement instruments comprised a study-developed sociodemographic questionnaire, the National Mental Health Literacy Questionnaire, the Patient Health Questionnaire-9 (PHQ-9), and the Generalized Anxiety Disorder Scale-7 (GAD-7). Multivariable logistic regression was used to identify factors associated with MHL, and Model 1 of the PROCESS macro for SPSS (with 5,000 bootstrap resamples) was used to test the moderating effect of MHL on the anxiety–depression association.

**Results:**

Only 22.4% of young adults had MHL attainment. Compared with participants without depressive or anxiety symptoms, those with such symptoms were less likely to have good MHL (both *p* < 0.05). Anxiety and depressive symptoms were strongly comorbid and positively correlated, and MHL significantly moderated this association (β = 2.04, *p* = 0.043). At low anxiety levels, the risk of depression was lower in the good MHL group than in the poor MHL group; at high anxiety levels, however, the rate of depression was higher in the good MHL group, suggesting that individuals with good MHL may identify their psychological issues earlier, resulting in a higher detection rate of depression during screening.

**Conclusion:**

MHL among Chinese young adults remains low and is closely linked to depressive and anxiety symptoms. For young adults with good MHL, early-screening pathways should be established to translate symptom awareness into timely professional assessment and intervention. For those without good MHL, public-health efforts should focus on improving symptom recognition and correcting cognitive biases that lead to underestimation of mental health risks and delays in help-seeking. however, this explanation remains speculative and requires further validation through longitudinal studies.

## 1 Introduction

### 1.1 Conceptual framework of mental health literacy

Mental health literacy (MHL) refers to an individual's ability to identify, manage, or prevent psychological disorders (1). Bjornsen et al. (2) further expanded this concept, defining it as the knowledge, attitudes, and skills required for individuals to acquire, maintain, and enhance positive mental health. This redefinition indicates that MHL should not be understood merely as the cognitive ability to “recognize diseases”, but rather as a comprehensive capability encompassing problem identification, emotional regulation, and health promotion.

### 1.2 MHL and symptoms of anxiety and depression

Depression and anxiety are common mental health disorders characterized by high prevalence rates and significant disease burden (3). Anxiety primarily manifests as excessive worry and tension, whereas depression is defined by persistent low mood and diminished interest in activities. The comorbidity between these conditions is widespread and can substantially exacerbate both the disease burden and functional impairment (4). Although evidence supports psychotherapy and pharmacotherapy as effective treatment modalities, their utilization remains limited (5). Studies have demonstrated that individuals often experience an anxiety response prior to developing a depressive state when confronted with stressful events (6, 7), indicating that anxiety serves more as an alarm signal for psychological distress and may function as a predictor of depression (8, 9). Failure to promptly regulate negative emotions increases susceptibility to anxiety, which subsequently precipitates depression and significantly elevates the risk of depressive episodes (7).

MHL plays a pivotal role in mental health promotion and disease prevention, and is closely associated with depressive and anxiety symptoms. High levels of MHL enable individuals to avoid unhealthy lifestyles that may trigger psychological distress (10–12), enhance early identification of psychological issues, and adopt proactive coping strategies and interventions to prevent further deterioration (13). Conversely, insufficient MHL impairs individuals' ability to recognize symptoms and reduces their willingness to seek professional help due to stigma (14). Delayed or inadequate seeking of assistance may lead to the progression of psychological problems into mental disorders, causing significant harm to both individual wellbeing and social functioning (15). Studies suggest that MHL may also influence the progression of negative emotions from mild discomfort to severe psychological disorders by enhancing individuals' cognitive evaluation of emotional symptoms and promoting positive coping mechanisms (16, 17). A recent meta-analysis further confirms that interactive, information and communication technology (ICT)-based interventions can effectively improve MHL, stigma-related attitudes, and help-seeking behaviors among adolescents and young adults (18).

Furthermore, emotional regulation strategies—particularly cognitive reappraisal and expressive inhibition—play a significant role in the development and comorbidity of social anxiety and depression (19), while coping styles have been demonstrated to mediate the transition from anxiety to depression (20). Notably, the latest evidence has begun to position MHL within the framework of anxiety and depression pathways, though it only explains the parent-child symptom associations between parental depression or anxiety and adolescent depression or anxiety symptoms in the form of a mediating variable (21). These findings suggest that MHL may shape the nature of the association between anxiety and depression. Currently, empirical studies directly examining the moderating effect of MHL in the anxiety-depression association remain limited [15; (42)], and the direction of this moderation remains unclear; thus, this study provides an exploratory investigation into this moderating effect.

### 1.3 Research background and hypotheses

Currently, China is undergoing a period of rapid socioeconomic transformation, characterized by an accelerated pace of life and intensified competitive pressures, with anxiety and depression-related issues drawing significant societal attention (22). The youth population (aged 18–35) is experiencing a critical developmental stage transitioning from late adolescence to adulthood, facing multiple challenges such as academic pressure, career transitions, and adaptation to social roles (23, 24). With the implementation of the Healthy China Initiative (2019–2030), mental health promotion has been incorporated into the national overall health strategy framework, making the mental health status of young people particularly worthy of greater attention. Therefore, we focus our analysis on this particular population. Based on the mentioned knowledge gaps, this study proposes three specific hypotheses: (1) The rate of youth meeting MHL standards is low and is influenced by sociodemographic characteristics; (2) MHL is significantly associated with anxiety and depressive symptoms, and individuals with adequate literacy have a lower risk of developing these symptoms; (3) MHL plays a moderating role in the association between anxiety and depressive symptoms.

## 2 Material and methods

### 2.1 Participants and procedure

According to Medium-and Long-Term Youth Development Plan in China (2016–2025), the target age range for youth is 14–35 years old. To comply with ethical requirements and explore the MHL characteristics of early adulthood, this study defines the young adults age range as 18–35 years old.

An anonymous cross-sectional study was conducted in Fujian by the Fujian Mental Health Center from September 2022 to February 2023. Multistage stratified cluster and random sampling method was applied to recruit the participants. The first stage sampled counties/districts, the second townships/streets; the third stage randomly sampled villages (rural areas) or residents committees (urban areas) in each township or street, as shown in Supplementary Figure S1.

The present analysis was not based on a sampling strategy specifically designed to recruit a separate representative youth adult sample. Rather, it was an age-restricted subgroup analysis derived from the broader general-population survey. A total of 13,000 individuals of the sampled villages were used as the samples, including 2,175 aged 18–35 years old. Participants with missing demographic information or incomplete responses to any key questionnaire required for the present analysis, including the Patient Health Questionnaire Depression Scale (PHQ-9), Generalized Anxiety Disorder Scale (GAD-7), or MHL questionnaire, were excluded (*n* = 11). Finally, 2,164 valid questionnaires were included in the analysis, with an average age of (29.49 ± 4.64) years. No item-level imputation was performed. The final analysis was based on complete cases only; all included participants had complete demographic information and complete PHQ-9, GAD-7, and MHL questionnaire data. A formal MCAR/MAR/MNAR assessment was not conducted because only 11 records were incomplete and the missingness involved key analytic variables required for score calculation.

### 2.2 Research tools

#### 2.2.1 Social and demographic questionnaire

The social demographic questionnaire used in this study is based on the survey instrument outlined in the “Notice of the Office of the China Health Action Promotion Committee on Issuing Interpretations of Key Indicators and Survey Methods for Mental Health Promotion Actions” (Guo Jian Tui Wei Ban Han [2020] No. 4). Sociodemographic data were collected using a study-specific demographic form developed by the research team to capture descriptive participants' information (age, gender, education level, place of residence, personal monthly income, household monthly income), behavioral habits (smoking, alcohol consumption), and chronic disease status over the past year. The detailed variables were defined as follows: (1) Occupation: mental work includes civil servants administrators, teachers, healthcare professionals, and other technical personnel; physical work includes farmers, workers, and military personnel; all other occupations are categorized as others. (2) Personal monthly income: low income ranges from 0 to 1,499 yuan; middle income from 1,500 to 7,999 yuan; high income >8,000 yuan. (3) Household monthly income: low income from 0 to 2,999 yuan; middle income from 3,000 to 9,999 yuan; high income >10,000 yuan. (4) Chronic disease status in the past year was analyzed as a binary variable. Participants who reported any chronic disease, including cardiovascular or cerebrovascular disease, digestive system disease, rheumatoid arthritis, herniated discs, chronic obstructive pulmonary disease, cancer, or other chronic conditions, were categorized as having chronic disease; those without any reported chronic disease were categorized as not having chronic disease.

#### 2.2.2 China resident MHL questionnaire

The Mental Health Literacy Questionnaire for China was employed to assess MHL, which is the designated monitoring tool when implementing the Mental Health Promotion Action, and consists of three sections: true/false questions, self-assessment questions, and case analysis questions. The true/false section comprises 20 items, each worth 5 points; correct answers receive 5 points, while incorrect answers or the “unknown” option receive 0 points (scoring range: 0–100). The self-assessment section includes 8 items, with scores ranging from 1 to 4 (total score: 8–32). The case analysis section consists of two sets of cases, each containing 4 items, with a total score range of 0–40. The definition of MHL attainment (Good MHL) is met when all three criteria are satisfied simultaneously: total true/false score ≥80, total self-assessment score ≥24, and total case analysis score ≥28. This scale has demonstrated good reliability and validity, with a Cronbach's α coefficient of 0.85 (25). In this study, the Cronbach's α coefficients for the true/false section were 0.806 and for the self-assessment section were 0.918.

#### 2.2.3 Patient health questionnaire depression scale (PHQ-9)

The PHQ-9 is a self-report scale designed to assess depressive symptoms over the past 2 weeks, comprising nine symptom items and one item evaluating overall functional impairment, with a total score range of 0 to 27 points. Scores of 0–4 indicate no depressive symptoms; 5–9 indicate mild depression; 10–14 indicate moderate depression; and ≥15 indicate severe depression. A total score of ≥10 indicates a positive screening result. Multiple studies have confirmed its good reliability and validity, with a Cronbach's α coefficient ranging from 0.90 to 0.91 (26, 27). The Cronbach's α coefficient was 0.959 in this study.

#### 2.2.4 Generalized anxiety disorder scale (GAD-7)

The GAD-7 is a self-report scale designed to assess anxiety symptoms over the past 2 weeks, comprising seven items with a total score range of 0–21 points. Anxiety severity is classified as clinically insignificant (0–4 points), mild (5–9 points), moderate (10–14 points), or severe (≥15 points). A total score ≥10 indicates a positive screening result. This scale has demonstrated excellent reliability and validity, with a Cronbach's α coefficient ranging from 0.93 to 0.94 (25, 28). The Cronbach's α coefficient was 0.954 in this study.

### 2.3 Statistical analysis

All analyses were performed using SPSS version 26.0. Categorical variables were expressed as frequencies and percentages; Between-group comparisons were performed using the chi-square test. Prior to regression analysis, a multicollinearity test was conducted on all independent variables, and results indicated no significant multicollinearity. Since all independent variables included in the regression model of this study are categorical variables, the assumption of a linear relationship between continuous independent variables and the logit function does not apply. Multivariate analysis employed a binary logistic regression model, with gender, smoking status, presence of chronic diseases, MHL attainment, depressive symptoms, and anxiety symptoms coded as dichotomous variables (0 = female /no /unattained /negative; 1 = male/yes/attained/positive), while marital status, educational level, occupation, personal monthly income, household monthly income, and alcohol consumption were included as dummy variables in the model. The moderating effect of MHL was tested using Model 1 from the SPSS process macro package (29) with 5,000 bootstrap resamples. All tests were two-sided, and *p* < 0.05 was considered statistically significant.

## 3 Results

### 3.1 Social demographic characteristics and distribution of MHL questionnaire survey results

In the MHL Questionnaire, the mean total score for true/false items was 65.07 ± 18.90, with 571 respondents (26.39%) achieving a score ≥80; the mean self-assessment item score was 28.38 ± 4.03, with 1,929 respondents (89.14%) scoring ≥24; the mean case analysis item score was 29.38 ± 7.65, with 1,315 respondents (60.77%) scoring ≥28; only 484 respondents (22.4%) met all three criteria, indicating that over 70% (77.6%) of the surveyed individuals failed to meet the required standards for MHL.

To compare the differences in general demographic characteristics between individuals with Good MHL and Poor MHL. The results indicate statistically significant differences between the two groups regarding education level (χ^2^ = 11.021, *p* = 0.012), marital status (χ^2^ = 9.437, *p* = 0.009), monthly personal income (χ^2^ = 9.382, *p* = 0.009), monthly household income (χ^2^ = 23.148, *p* < 0.001), alcohol consumption (χ^2^ = 20.331, *p* < 0.001), smoking status (χ^2^ = 35.289, *p* < 0.001), prevalence of chronic diseases (χ^2^ = 4.812, *p* = 0.028), depressive symptoms (χ^2^ = 12.283, *p* < 0.001), and anxiety symptoms (χ^2^ = 16.804, *p* < 0.001), whereas no significant differences were observed for gender or occupation (Table 1).

**Table 1:** The univariate analysis for MHL among youth adults [*n* (%)].

Variables	All population (*n* = 2,164)	Good MHL		χ^2^	*p*
		No (*n* = 1,680)	Yes (*n* = 484)		
Gender					
Male	1,047 (48.4)	819 (48.75)	228 (47.11)	0.406	0.524
Female	1,117 (51.6)	861 (51.25)	256 (52.89)		
Education levels					
Primary school and below	119 (5.5)	106(6.31)	13 (2.69)	11.021	0.012
Middle school	596 (27.5)	466 (27.74)	130 (26.86)		
High school	578 (26.7)	435 (25.89)	143 (29.55)		
College and above	871 (40.2)	673 (40.06)	198 (40.91)		
Occupation					
Mental work	567 (26.2)	441 (26.25)	126 (26.03)	2.775	0.250
Physical work	645 (29.8)	514 (30.6)	131 (27.07)		
Others	952 (44.0)	725 (43.15)	227 (46.90)		
Marital status					
Unmarried	752 (34.8)	558 (33.21)	194 (40.08)	9.437	0.009
Married	1,348 (62.3)	1,067 (63.51)	281 (58.06)		
Others	64 (3.0)	55 (3.27)	9 (1.86)		
Personal monthly income					
Low	434 (20.1)	357 (21.25)	77 (15.91)	9.382	0.009
Middle	1,610 (74.4)	1,224 (72.86)	386 (79.75)		
High	120 (5.5)	99 (5.89)	21 ( 4.34)		
Household monthly income					
Low	228 (10.5)	198 (11.79)	30 (6.2)	23.148	< 0.001
Middle	985 (45.5)	784 (46.67)	201 (41.53)		
High	951 (43.9)	698 (41.55)	253 (52.27)		
Alcohol consumption					
No	1,700 (78.6)	1,284 (76.43)	416 (85.95)	20.331	< 0.001
Former	68 (3.1)	57 (3.39)	11 (2.27)		
Current	396 (18.3)	339 (20.18)	57 (11.78)		
Smoking					
No	1,785 (82.5)	1342(79.88)	443(91.53)	35.289	< 0.001
Yes	379 (17.5)	338(20.12)	41( 8.47)		
Chronic disease status					
No	1,646 (76.1)	1,296 (77.14)	350 (72.31)	4.812	0.028
Yes	518 (23.9)	384 (22.86)	134 (27.69)		
Depressive symptoms					
No	2,052 (94.82)	1,578 (93.93)	474 (97.93)	12.283	< 0.001
Yes	112 (5.18)	102 (6.07)	10 (2.07)		
Anxiety symptoms					
No	2,055 (94.96)	1,578 (93.93)	477 (98.55)	16.804	< 0.001
Yes	109 (5.04)	102 (6.07)	7 (1.45)		

### 3.2 Analysis of the factors influencing MHL attainment

Using the attainment or not of MHL as the dependent variable, nine variables with *p* < 0.05 from the univariate analysis were included in the multivariate logistic regression model.

For the multivariate Logistic regression model examining factors influencing MHL attainment, the Hosmer–Lemeshow (H–L) test yielded a χ^2^ value of 20.732 (*p* = 0.008), indicating poor model fit. Further evaluation using the ROC curve revealed an area under the curve (AUC) of 0.659 with a 95% confidence interval of 0.631–0.687, suggesting moderate discriminative power overall. After excluding educational level and alcohol consumption status, the H–L test demonstrated good model fit (χ^2^ = 15.093, *p* = 0.057). Given that the H–L test is highly sensitive to minor deviations in large sample sizes, and that educational level and alcohol consumption exhibited statistical significance in the univariate analysis while being theoretically closely associated with mental health literacy, the primary objective of this model was to identify associated factors rather than provide individual-level predictions. Removal of these two variables improved model fit but may lead to loss of critical information. Consequently, the original model was retained in this study.

After controlling for confounding factors, the results showed (Table 2): In terms of educational level, individuals with junior high school education (β = 0.70, OR = 2.01,95% CI: 1.08–3.75, *p* = 0.03) and high school/technical secondary education (β = 0.75, OR = 2.13,95% CI: 1.14–3.97, *p* = 0.02) achieved higher rates of MHL attainment compared to those with primary school education or below; however, no statistically significant difference was observed between these groups and those with college education or above (β = 0.55, OR = 1.73, 95% CI: 0.93–3.22, *p* = 0.08). Regarding marital status, married individuals exhibited lower MHL attainment rates than unmarried individuals (β = −0.35, OR = 0.70, 95% CI: 0.56–0.88, *p* < 0.001). In terms of economic income, individuals with moderate personal income (β = 0.34, OR = 1.41, 95% CI: 1.04–1.91, *p* = 0.03) and those from high-income households (β = 0.71, OR = 2.04, 95% CI: 1.29–3.22, *p* < 0.001) demonstrated higher attainment rates. For health behaviors, smokers showed significantly lower attainment rates compared to non-smokers (β = −0.87, OR = 0.42, 95% CI: 0.29–0.61, *p* < 0.001). Regarding health status, the attainment rates was higher among patients with chronic diseases compared to those without chronic diseases (β = 0.41, OR = 1.51, 95% CI: 1.18–1.93, *p* < 0.001). Patients with depressive symptoms (β = −0.74, OR = 0.48, 95% CI: 0.23–0.98, *p* = 0.04) and those with anxiety symptoms (β = −1.14, OR = 0.32, 95% CI: 0.14–0.73, *p* = 0.01) exhibited significantly lower attainment rates than asymptomatic individuals.

**Table 2:** The multivariate logistic regression analysis for MHL among youth adults.

Variables	β	*S.E*.	*Wald*	*p*	*OR*	95% CI
Education level (Ref. Primary school and below)						
Middle school	0.70	0.32	4.84	0.03	2.01	(1.08, 3.75)
High school	0.75	0.32	5.62	0.02	2.13	(1.14, 3.97)
College and above	0.55	0.32	3.03	0.08	1.73	(0.93, 3.22)
Marital status (Ref. Unmarried)						
Married	−0.35	0.12	9.16	0.00	0.70	(0.56, 0.88)
Others	−0.68	0.38	3.13	0.08	0.51	(0.24, 1.08)
Personal monthly income (Ref. Low)						
Middle	0.34	0.16	4.82	0.03	1.41	(1.04, 1.91)
High	−0.10	0.30	0.11	0.75	0.91	(0.51, 1.62)
Household monthly income (Ref. Low)						
Middle	0.41	0.23	3.11	0.08	1.50	(0.96, 2.37)
High	0.71	0.23	9.29	0.00	2.04	(1.29, 3.22)
Smoking (Ref. No)						
Yes	−0.87	0.19	20.35	0.00	0.42	(0.29, 0.61)
Chronic disease status (Ref. No)						
Yes	0.41	0.12	11.00	0.00	1.51	(1.18, 1.93)
Alcohol consumption (Ref. No)						
Former	−0.38	0.35	1.18	0.28	0.69	(0.35, 1.35)
Yes	−0.33	0.17	3.74	0.05	0.72	(0.51, 1.00)
Depression (Ref. No)						
Yes	−0.74	0.36	4.12	0.04	0.48	(0.23, 0.98)
Anxiety (Ref. No)						
Yes	−1.14	0.42	7.28	0.01	0.32	(0.14, 0.73)

### 3.3 The moderating role of MHL in the association between anxiety and depression

This study employed the chi-square test to compare differences in MHL attainment and anxiety symptoms between participants with and without depressive conditions. As shown in Table 3, the proportion of participants with depressive states non-attainment was as high as 91.1%, significantly higher than that among non-depressive participants (76.9%; χ^2^ = 12.280, *p* < 0.001). Additionally, 50.00% of individuals with depressive states exhibited positive anxiety symptoms, compared to only 2.60% in non-depressive participants, a highly significant difference (χ^2^ = 499.212, *p* < 0.001). These results indicate a significant association among MHL, anxiety disorders, and depression.

**Table 3:** The comparison between depression, MHL, and anxiety among youth adults [*n*(%)].

Variables	All population (*n* = 2,164)	Depression (*n* = 112)	Non-depression (*n* = 2,052)	χ^2^	*p*
Good MHL					
No	1,680 (77.6)	102 (91.1)	1,578 (76.9)	12.280	< 0.001
Yes	484 (22.4)	10 (8.9)	474 (23.1)		
Anxiety symptoms					
No	2,055 (94.96)	56 (50.00)	1,999 (97.4)	499.212	< 0.001
Yes	109 (5.04)	56 (50.00)	53 (2.6)		

To further examine whether MHL exerts a moderating effect on the association between anxiety and depression, this study employed the SPSS PROCESS 4.1 macro program (Model 1) for moderation analysis. This model utilized anxiety symptoms as the independent variable, depressive symptoms as the dependent variable, and whether MHL attainment as the moderating variable. Gender, marital status, educational level, occupation, monthly personal income, monthly household income, history of chronic diseases, alcohol consumption, and smoking status were controlled as covariates. The VIF values ranged from 1.027 to 1.489 with a tolerance range of 0.672–0.974, significantly below the commonly accepted thresholds of VIF < 5/10 and Tolerance > 0.1/0.2, indicating no significant multicollinearity. Regarding the moderating effect of MHL on the association between anxiety and depression, the H–L test showed a χ^2^ value of 12.867 (*p* = 0.117), indicating good model fit.

The effects of anxiety on depression and the moderating effect of MHL were estimated within a binary logistic regression framework, with all statistical analyses performed using bias-corrected Bootstrap sampling (5,000 replicates). The results are presented in Table 4: Anxiety symptoms exhibited a significant positive predictive effect on depressive symptoms (β = 3.34, OR = 28.12, 95% CI: 16.83–47.08, *p* < 0.001); MHL attainment had a significant negative impact on depression (β = −1.11, OR = 0.33,95% CI: 0.13–0.84, *p* = 0.020); and the interaction term between anxiety symptoms and MHL was statistically significant (β = 2.04, OR = 7.64,95% CI: 1.07–54.60, *p* = 0.043), indicating that MHL significantly moderates the relationship between anxiety and depression. In addition to statistical significance, the OR (OR = 7.64, 95% CI: 1.07–54.60, *p* = 0.043) for the interaction term was reported between anxiety symptoms and MHL. This interaction term OR indicates that among individuals with positive anxiety symptoms, those with adequate MHL had a depression incidence OR approximately 7.64 times higher than those without adequate literacy; in terms of predictive probability, the difference in depression probability between the two groups was 22.55 percentage points (65.73% vs. 43.18%), suggesting that the moderation effect has clinical significance.

**Table 4:** The moderating role of MHL in the association between anxiety and depressio*n* (*N* = 2,164).

Variables	Depression			
	β	*p*	OR	95% CI
Gender	0.50	0.07	1.65	(0.95–2.83)
Education levels	0.26	0.06	1.29	(0.99–1.68)
Occupation	0.05	0.74	1.05	(0.79–1.39)
Marital status	−0.50	0.02^*^	0.60	(0.39–0.93)
Personal monthly income	−0.47	0.02^*^	0.63	(0.43–0.92)
Household monthly income	−0.47	0.02^*^	0.63	(0.43–0.92)
Alcohol consumption	0.27	0.08	1.31	(0.97–1.77)
Smoking	0.27	0.43	1.31	(0.67–2.57)
Chronic disease status	1.03	< 0.001^***^	2.81	(1.76–4.47)
Anxiety risk	3.34	< 0.001^***^	28.12	(16.83–47.08)
Good MHL	−1.11	0.02^*^	0.33	(0.13–0.84)
Anxiety risk ^*^ Good MHL	2.04	0.04^*^	7.64	(1.07–54.60)

^*^p < 0.05, ^**^p < 0.01, ^***^p < 0.001.

To more intuitively illustrate the moderating effect of MHL, a simple slope analysis plot was further constructed (Figure 1). In the group with MHL non-attainment, the predictive effect of anxiety on depression was 3.34 (*p* < 0.001); in the group with MHL attainment, this effect was 5.38 (*p* < 0.001), and the difference in slopes between the two groups was statistically significant (*p* = 0.043). Further calculations of depression probability revealed that, in the absence of anxiety risk, the depression probabilities were 2.63% and 0.88% in the MHL non-attainment and MHL attainment groups, respectively, with the MHL attainment group exhibiting a lower depression probability; in the presence of anxiety risk, the depression probabilities were 43.18% and 65.73% in both groups, respectively, with the MHL attainment group showing a higher depression probability. Regardless of whether MHL attainment or not, an increase in anxiety risk was associated with a significant rise in depression risk. However, the upward trend was significantly more pronounced in the MHL attainment group compared to the MHL non-attainment group.

**Figure 1:**
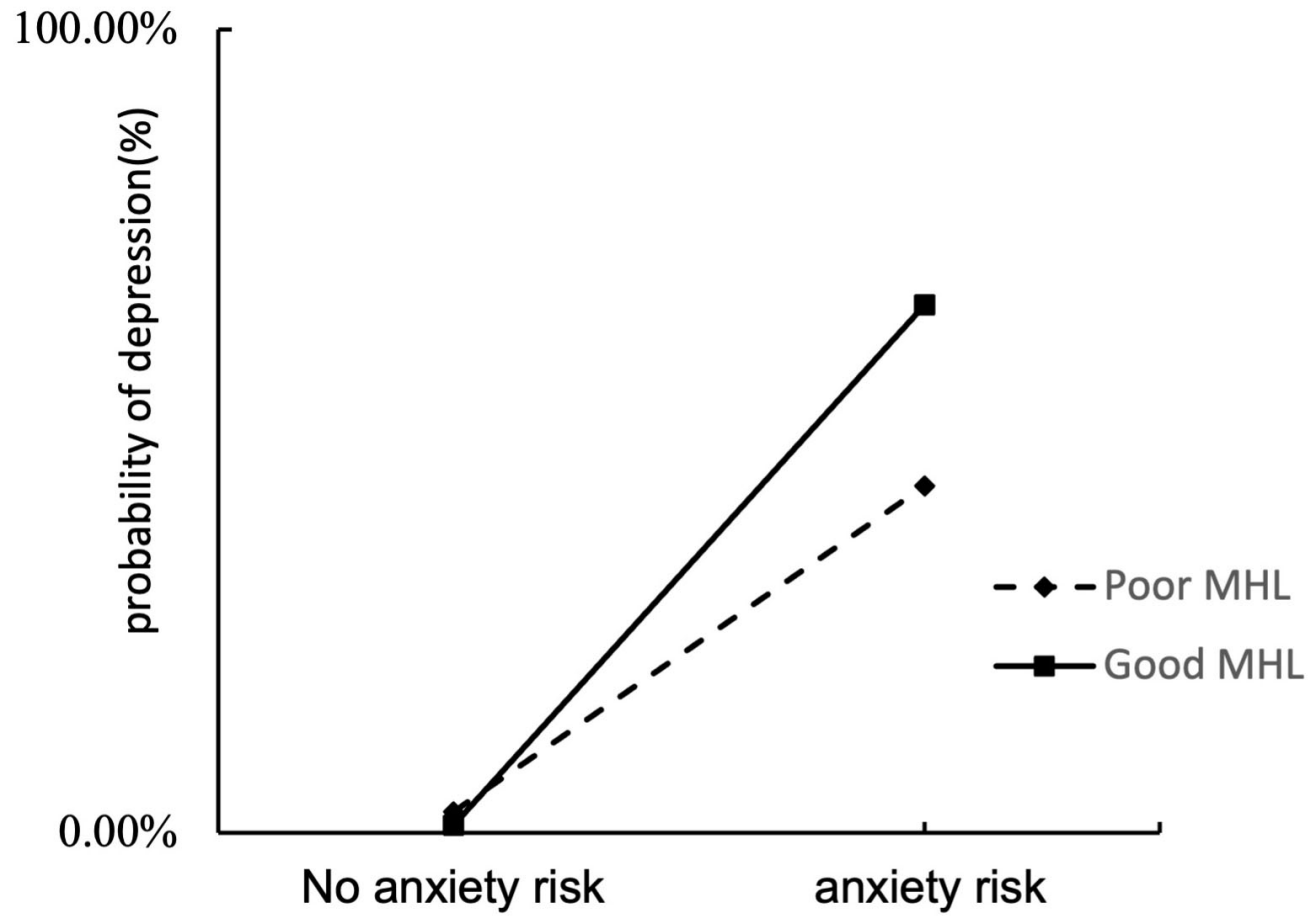
A simple slope analysis for the moderation effect of MHL.

## 4 Discussion

### 4.1 Current status and influencing factors of MHL among young adults

The survey results of this study indicate that the attainment rate of MHL among young adults in Fujian Province is 22.4%, which is consistent with previous findings, suggesting that young adults exhibit a low to moderate level of MHL (30, 31). While this meets the goal set by the Healthy China Initiative (2019–2030) of “raising the MHL attainment of residents to 20% by 2022,” it still falls short of the initiative's broader target of “ensuring that the national MHL attainment reaches at least 30% by 2030.” Additionally, the study revealed significant disparities in the attainment rates across various dimensions of the MHL questionnaire, indicating that although young adults can accurately assess their own psychological states and demonstrate strong self-awareness and social expectation tendencies, their comprehensive understanding of mental health knowledge remains inadequate.

The study results demonstrate that educational level, marital status, and monthly personal/family income independently and significantly influence the attainment rate of MHL among young adults. Regarding education level, individuals with junior high school or high school/technical secondary education demonstrated significantly higher MHL attainment rates compared to those with primary school education or below, indicating that acquiring and mastering mental health knowledge requires a certain level of education. Young adults with higher educational backgrounds typically possess stronger information acquisition and analysis skills, enabling them to proactively access relevant information and thereby enhance their MHL (32, 33). In terms of marital status and income, young adults from high-income families, those with moderate monthly incomes, and unmarried individuals exhibited greater advantages in achieving MHL goals, likely due to their relatively favorable living conditions that facilitate access to diverse sources of mental health knowledge (34). Regarding health status, the MHL attainment rate among individuals with chronic diseases was 25.87%, exceeding the average, suggesting heightened concern of health-related issues within this population.

Therefore, the MHL attainment is influenced by a combination of demographic characteristics, socioeconomic factors, and health status. When implementing interventions, particular attention should be paid to economically underdeveloped regions, low-income populations, and individuals with lower educational levels, by enhancing cognitive reserves through educational investment and improved accessibility to mental health services.

### 4.2 The moderating effect of MHL on anxiety and depressive symptoms

This study found that the detection rates of anxiety and depression symptoms among young adults were 5.04% and 5.18%, respectively, which are close to the national prevalence rate of anxiety symptoms of 5.0% reported in the China Mental Health Survey (CMHS), but significantly higher than the prevalence rate of depression symptoms at 3.4% (22). Regardless of whether MHL attainment or not, an increased risk of anxiety was accompanied by a significant rise in the risk of depression. This indicates a comorbidity phenomenon between anxiety and depression, consistent with previous research findings (7).

This study also found that individuals exhibiting depressive or anxiety symptoms had a significantly higher proportion of those with MHL non-attainment compared to those without such symptoms, consistent with previous findings (12, 25). When individuals with MHL attainment, their risk of developing depression or anxiety is correspondingly reduced. This is likely due to the heightened awareness of their own psychological states (e.g., anxiety, low mood) among individuals with higher MHL (35). They are able to identify early signs of depression and seek timely assistance or evaluation, thereby facilitating earlier detection of mental health issues such as anxiety and depression, which helps mitigate psychological distress effectively (36). Conversely, individuals with MHL non-attainment are more prone to adopting unhealthy lifestyle behaviors as self-help measures (37, 38); for instance, smokers in this study demonstrated a 58% lower probability of with MHL attainment compared to non-smokers, with the rate of MHL attainment only 10.82%, significantly below the average level. Additionally, MHL non-attainment may lead to delayed intervention due to difficulties in symptom recognition, further increasing the risk of worsening depressive or anxiety symptoms (25).

This finding can be interpreted from multiple perspectives. First, from the perspective of cognitive appraisal, good MHL helps individuals more accurately identify negative emotional experiences such as anxiety and depression, thereby enabling them to adopt proactive coping strategies (such as cognitive reappraisal and problem-solving) earlier on, rather than maladaptive emotional regulation methods (such as expression suppression and rumination), which in turn helps prevent the progression of anxiety to depression (19, 35). Second, from the perspective of behavioral coping, individuals with good MHL are more likely to view psychological distress as a signal that professional help is needed, thereby proactively seeking social support or professional services to prevent the problem from worsening (13, 14). However, when anxiety is already at a high level, the heightened self-awareness and symptom sensitivity associated with MHL may also make individuals more likely to recognize and report their own depressive symptoms, resulting in “higher detection rates” in cross-sectional surveys. This does not imply that MHL causes depression; rather, it likely reflects that literacy facilitates the earlier and more accurate identification of underlying issues. Furthermore, even individuals with good MHL who are experiencing high levels of anxiety may find it difficult to effectively apply their knowledge and skills due to severe emotional distress, or may be constrained by factors such as stigma, preventing them from translating their cognitive advantages into help-seeking behaviors, which leads to the persistence of symptoms. Therefore, From the perspectives of cognitive appraisal and coping theory, as well as the stress-buffering hypothesis, the moderating effect of mental health literacy on the anxiety–depression association is not unidirectional protection; rather, it may involve two mechanisms-“protective buffering” and “sensitivity amplification”-each dominating under different levels of anxiety: the former is more likely to play a dominant role at low anxiety levels, whereas the latter becomes relatively more prominent at high anxiety levels due to enhanced symptom recognition coupled with limited coping capabilities (39–41). Since this study employs a cross-sectional design, this interpretation is merely speculative and does not rule out symptom reporting bias, reverse causality, or other alternative explanations.

This study found that the relationship between anxiety and depression is moderated by MHL. Individuals with MHL attainment exhibit lower depressive risk even at low anxiety levels, but conversely, higher depressive risk occurs at elevated anxiety levels. This may be because individuals with MHL attainment are more inclined to acknowledge their psychological issues, resulting in higher statistical detection rates of depression. This does not imply that MHL directly increases depressive risk; rather, it enables earlier identification of such issues.

### 4.3 Limitation

Several limitations should be acknowledged. First, although the parent survey used a province-wide stratified multistage sampling design, it was designed to represent the general adult population rather than young adults specifically. The present study analyzed an age-restricted subgroup of participants aged 18–35 years, and this subgroup may not fully reflect the demographic structure of the target young adult population in Fujian Province. Therefore, the findings should be interpreted as associations observed among young adult respondents derived from the parent survey, and their generalizability to all young adults in Fujian Province should be interpreted with caution. Second, the de-identified dataset lacked sampling weights, stratum identifiers, and cluster identifiers, preventing full adjustment for the complex survey design and potentially leading to underestimated standard errors. Third, no formal *a priori* sample size calculation was conducted for the present age-restricted analysis. Although the overall sample size was adequate relative to the number of covariates in the adjusted models, subgroup and interaction analyses may still have had limited precision and should be interpreted cautiously. Fourth, 11 participants with missing basic information or incomplete PHQ-9, GAD-7, or MHL questionnaire data were excluded, and complete-case analysis may have introduced selection bias. Fifth, chronic disease status was analyzed as a binary variable, and the dataset did not include detailed information on pre-existing psychiatric disorders, epilepsy, neurological diseases, or ongoing psychiatric treatment; therefore, disease-specific effects and potential residual confounding by severe psychiatric or neurological conditions could not be assessed. Sixth, the cross-sectional design precludes causal inference and assessment of symptom recognition time, prior diagnosis, mental health service use, or previous mental health education; longitudinal studies are needed to clarify temporal relationships among mental health literacy, psychological symptoms, help-seeking behaviors, and clinical outcomes. Seventh, defining good MHL as simultaneous fulfillment of all three criteria may have masked heterogeneity across literacy dimensions and may be influenced by overconfidence or social expectation bias. Eighth, the subgroup with positive depressive symptoms was small, resulting in sparse cross-cell counts and an imprecise interaction estimate; larger studies are needed to validate the observed moderation effect. Finally, although the analyses adjusted for multiple variables, residual confounding may remain. Unmeasured factors, including personality traits such as neuroticism, adverse childhood experiences, social support, genetic susceptibility, and prior use of mental health services, may influence both MHL and susceptibility to anxiety and depressive symptoms. Because these factors were not captured in the present study, we cannot fully exclude the possibility that unmeasured confounding biased the observed associations. Future studies should incorporate more comprehensive psychosocial and biological measures to better estimate the relationship between MHL and emotional symptoms.

### 4.4 Conclusion

The study findings reveal that the MHL attainment rate among young adults aged 18–35 remains low, with significantly lower proportions of individuals exhibiting depressive or anxious symptoms with MHL attainment compared to those without such symptoms. MHL may play a moderating role in the association between anxiety and depressive symptoms. MHL exerts a moderating effect on anxiety and depressive symptoms: it may be associated with anxiety to depression by fostering positive coping strategies, or facilitate early detection of underlying issues by enhancing symptom recognition sensitivity. Therefore, for individuals with MHL attainment, early screening mechanisms should be established to leverage their symptom identification strengths for timely professional assessment and intervention; for those individuals with MHL non-attainment, efforts should focus on improving MHL levels, refining symptom recognition skills, and correcting cognitive biases that lead to underestimated health risks and delayed seeking of help. It should be emphasized that, given the cross-sectional design, these interpretations represent speculative mechanisms; this study can only demonstrate correlations between variables and cannot establish causal relationships. Futural research should further examine the relationship between MHL and emotional distress through longitudinal studies, while developing targeted interventions to enhance MHL among young adults and promote their overall wellbeing.

## Data Availability

The data that support the findings of this study are available from the corresponding author upon reasonable request.
