# Supplementary material for: Mental health literacy moderates the association between anxiety and depressive symptoms among Chinese young adults: a population-based cross-sectional study
Source: Front Public Health. 2026 Oct 16;14:1872627. doi: 10.3389/fpubh.2026.1872627 (PMC13721286; doi:10.3389/fpubh.2026.1872627)
Supplement: Supplementary file 1 [file Table_1.docx]

**Supplementary Table**

**Supplementary Table S1.** Cross-tabulation of depression risk, anxiety risk, and MHL status (n = 2,164)

| Depression risk | Anxiety risk: Yes | | Anxiety risk: No | | Total | |
| --- | --- | --- | --- | --- | --- | --- |
|  | poor MHL | good MHL | poor MHL | good MHL | poor MHL | good MHL |
| Yes  (n=112) | 51 | 5 | 51 | 5 | 102 | 10 |
| No (n=2052) | 51 | 2 | 1527 | 472 | 1578 | 474 |
| Total | 102 | 7 | 1578 | 477 | 1680 | 484 |
